# Development and validation of a nomogram based on tumor margin irregularity and alpha-fetoprotein for predicting microvascular invasion in hepatocellular carcinoma

**DOI:** 10.3389/fonc.2026.1821034

**Published:** 2026-06-26

**Authors:** Xiang Huang, Xiaofeng Chen, Xiangguang Chen, Ruibin Huang, Zhuozhi Dai, Ruyao Zhuang, Zhiqi Yang

**Affiliations:** 1 Department of Radiology, Meizhou People’s Hospital, Meizhou, China; 2 Department of Radiology, First Affiliated Hospital of Shantou University Medical College, Shantou, China; 3 Department of Radiology, Shantou Central Hospital, Shantou, Guangdong, China

**Keywords:** alpha-fetoprotein, computed tomography, hepatocellular carcinoma, microvascular invasion, nomogram

## Abstract

**Objective:**

To develop and externally validate a preoperative nomogram based on tumor margin irregularity and alpha-fetoprotein (AFP) positivity for predicting microvascular invasion (MVI) in hepatocellular carcinoma (HCC).

**Methods:**

This retrospective multicenter study included a training cohort of 487 patients and an external validation cohort of 256 patients with pathologically confirmed HCC after liver resection. Demographic, clinical, tumor-related, serological, and preoperative computed tomography (CT) variables were compared by MVI status. Two blinded radiologists independently assessed qualitative CT features, and interobserver agreement was evaluated using Cohen’s κ. Least Absolute Shrinkage and Selection Operator (LASSO) regression with 10-fold cross-validation was used for predictor selection, followed by multivariable logistic regression. Model performance was assessed by receiver operating characteristic (ROC) curves, area under the curve (AUC), DeLong tests, calibration, the Hosmer–Lemeshow test, and decision curve analysis (DCA). Tumor size-related sensitivity analyses were performed.

**Results:**

MVI was present in 187 of 487 patients in the training cohort and 95 of 256 patients in the validation cohort. LASSO selected AFP positivity, irregular tumor margins, capsular interruption, and intratumoral hyperplastic vessels. Multivariable analysis identified irregular tumor margins (adjusted odds ratio [OR] = 5.275, 95% confidence interval [CI]: 3.165–8.791, p < 0.001) and AFP positivity (adjusted OR = 3.297, 95% CI: 1.983–5.481, p < 0.001) as independent predictors. The final two-variable model achieved AUCs of 0.740 and 0.781 in the training and validation cohorts. Tumor size did not materially improve discrimination, and model performance was generally consistent across size categories. Calibration was acceptable, and DCA suggested exploratory clinical utility.

**Conclusion:**

The nomogram based on tumor margin irregularity and AFP positivity showed fair-to-acceptable discrimination and may support exploratory preoperative MVI risk stratification in surgically treated or resectable HCC patients with clinical characteristics similar to those in the study cohorts, pending prospective multicenter validation.

## Introduction

Hepatocellular carcinoma (HCC) is one of the most common and lethal malignancies worldwide and accounts for most primary liver cancers. According to global cancer statistics, primary liver cancer remains among the leading causes of cancer-related mortality, with a particularly high disease burden in regions with prevalent chronic hepatitis B virus (HBV) infection, including China (1). For patients with early-stage or resectable HCC, liver resection, local ablation, and liver transplantation remain potentially curative treatments, and treatment selection should consider tumor burden, liver function, portal hypertension, performance status, and expected oncologic benefit (2, 3). However, postoperative recurrence remains a major obstacle after curative-intent treatment. In HCC-specific studies, recurrence risk has been associated with tumor-related factors such as tumor size, tumor number, alpha-fetoprotein (AFP) level, and vascular invasion, highlighting the need for improved preoperative risk stratification (4).

Microvascular invasion (MVI) is an important histopathological marker of aggressive HCC biology. It is generally defined as microscopic tumor invasion within vascular spaces lined by endothelial cells and can only be confirmed by pathological examination after resection or transplantation. The severity and extent of MVI have been shown to correlate with prognosis and imaging findings, supporting its role as a clinically relevant marker rather than a purely pathological descriptor (5). Because MVI is not directly available before surgery, preoperative identification of patients at increased risk of MVI may provide supportive information for multidisciplinary discussion, surgical planning, resection margin assessment, and postoperative surveillance. Nevertheless, MVI prediction should be regarded as risk stratification rather than a substitute for individualized clinical judgment.

Several preoperative models have been proposed for MVI prediction in HCC. Many recent studies have focused on magnetic resonance imaging (MRI) radiomics, computed tomography (CT) radiomics, or machine-learning approaches, which can extract high-dimensional quantitative information from medical images and may improve risk prediction (6, 7). However, radiomics-based models often require tumor segmentation, feature engineering, scanner harmonization, local validation, and technical expertise, which may limit their immediate use in routine clinical practice. In parallel, non-radiomics nomograms based on clinical laboratory indices and conventional radiological findings have also been developed, but many of these models remain limited by single-center design, incomplete external validation, inconsistent variable selection, or insufficient comparison with simpler individual predictors (8, 9). Therefore, there remains a practical need for an interpretable model based on routinely available preoperative variables.

The present study was designed within this context. Rather than proposing a complex radiomics or deep-learning model, we aimed to develop and externally validate a simple, non-radiomics nomogram using conventional CT imaging features and routine serological markers. We specifically evaluated whether combining visually assessable CT features with AFP status could provide reproducible and clinically interpretable information for preoperative MVI risk stratification. This study also incorporated LASSO-based feature selection, multivariable logistic regression, external validation, calibration assessment, DeLong comparison of AUCs, and decision curve analysis to more comprehensively evaluate model performance and potential clinical utility. Accordingly, the objective of this study was to develop and externally validate an exploratory preoperative nomogram integrating conventional CT features and routine serum markers for predicting MVI in patients with HCC.

## Materials and methods

### Patients

This retrospective multicenter study included a training cohort and an independent external validation cohort of patients with pathologically confirmed HCC who underwent preoperative CT and three-phase contrast-enhanced scans. To establish the training cohort, we consecutively evaluated patients who underwent curative-intent liver resection at Meizhou People’s Hospital between September 2015 and December 2021. After applying the strict inclusion and exclusion criteria, 487 eligible patients (187 MVI-positive and 300 MVI-negative) were enrolled. To evaluate the generalizability of the proposed predictive model, an independent external validation cohort comprising 256 patients (95 MVI-positive and 161 MVInegative) was retrospectively established from two independent institutions (First Affiliated Hospital of Shantou University Medical College and Shantou Central Hospital) between January 2018 and December 2024. The study protocol was approved by the Medical Ethics Committees of all participating institutions, including the primary institutional review board at Meizhou People’s Hospital (No. 2024-C-136). Written informed consent was obtained from all participating patients prior to treatment.

**Figure 1:**
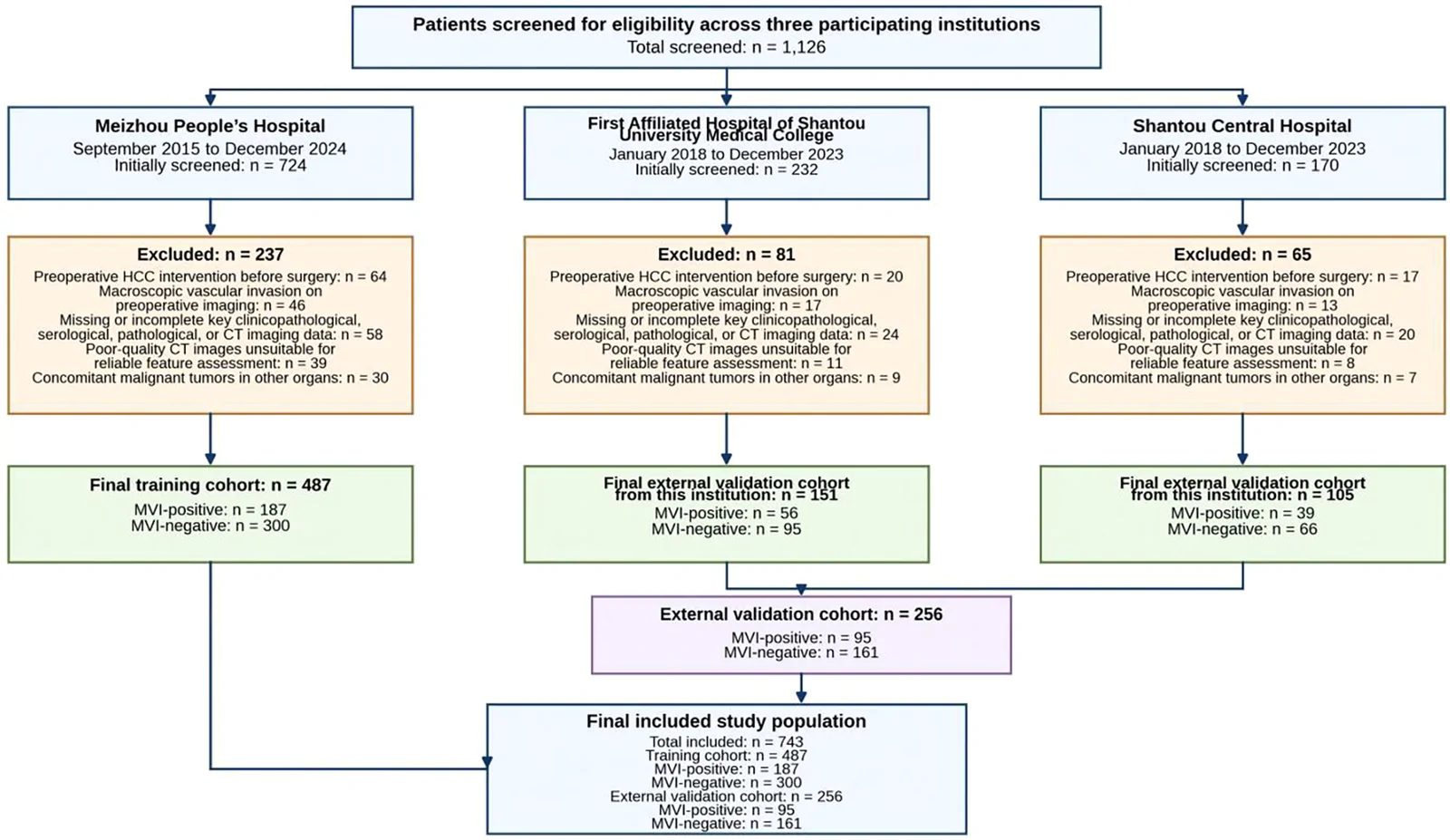
Patient enrollment flow diagram. The diagram shows the number of patients initially screened at each participating institution, the number excluded according to each predefined exclusion criterion, and the final numbers included in the training and external validation cohorts stratified by microvascular invasion (MVI) status. HCC, hepatocellular carcinoma; CT, computed tomography; MVI, microvascular invasion.

The detailed patient screening process at each participating institution, including the number of initially screened patients, exclusions according to predefined criteria, and final cohort composition stratified by MVI status, is shown in **Figure 1**.

### Criteria for inclusion and exclusion

The inclusion criteria were as follows: (1) pathologically confirmed HCC after curative-intent liver resection; (2) available postoperative histopathological assessment of MVI status; (3) preoperative plain and three-phase contrast-enhanced CT of the upper abdomen or whole abdomen performed before surgery; and (4) complete clinical, serological, pathological, and CT imaging data required for model development and validation.

Exclusion criteria were applied to the study population, including the following: (1) patients who had undergone preoperative HCC intervention; (2) patients with macroscopic vascular invasion, such as portal vein or hepatic vein tumor thrombosis, identified on preoperative imaging; (3) patients with missing or incomplete key clinicopathological, serological, pathological, or CT imaging data required for model development and validation; (4) individuals with poor-quality CT images unsuitable for reliable feature assessment; and (5) subjects with concomitant malignant tumors in other organs.

### Pathological diagnostic criteria for MVI

The pathological diagnosis of MVI was determined based on the post-operative histopathological examination of the resected liver specimens. Routine sampling included a 7-point baseline sampling method from the tumor and adjacent liver tissues. The specimens were fixed in 10% neutral buffered formalin, embedded in paraffin, and subjected to standard hematoxylin and eosin (H&E) staining. MVI was strictly defined microscopically as the presence of cancer cell nests within the vascular lumen (including small branches of the portal vein, hepatic vein, or tumor capsular vessels) lined by endothelial cells. To ensure diagnostic accuracy, all pathological slides were independently reviewed by two hepatobiliary pathologists with over 10 years of experience, who were blinded to the patients’ clinical and imaging data. Any diagnostic discrepancies were resolved through consensus discussion.

### CT acquisition protocol

All patients fasted for 4 to 8 hours and consumed 200 ml of water before the examination. The patients were positioned supine for CT imaging using a Siemens third-generation dual-source Force CT scanner to capture images of the entire liver. The scanning parameters included a slice thickness of 0.625 mm, tube voltage of 120 kV, and automatic tube current. Subsequently, patients received an intravenous injection of 300 mg/ml iohexol at a dose of 1.5 ml/kg for contrast-enhanced scanning. The parameters for the contrast-enhanced scan were a slice thickness and interval of 6 mm, voltage of 120 kV, automatic tube current, and a matrix size of 512×512. Contrast enhancement was triggered by monitoring the abdominal aorta at the level of the hepatic hilum, with a CT threshold of 100 HU. Scans were performed during the arterial phase, portal venous phase, and delayed phase at 25–30 seconds, 50–65 seconds, and 180 seconds post-triggering, respectively. After image acquisition, anonymized CT images were transferred to a dedicated workstation and independently reviewed by two radiologists with more than 6 years of abdominal imaging experience. During image review, both radiologists were blinded to all clinical, laboratory, pathological, and follow-up information, including preoperative AFP levels, final pathological MVI status, treatment information, and postoperative clinical outcomes. Any discrepancies in qualitative imaging features, including tumor margin assessment, capsular interruption, and intratumoral hyperplastic vessels, were resolved by consensus discussion without access to clinical or pathological data. Image analysis utilized a window width of 250 HU and a window level of 40 HU.

### Observation indicators

Clinical demographic variables, including age and sex, and viral hepatitis status, including hepatitis B surface antigen (HBsAg) positivity and anti-hepatitis C virus (HCV) antibody positivity, were systematically extracted from electronic medical records. Cirrhosis status was determined according to clinical records, laboratory findings, and imaging evidence documented before surgery (2). Liver functional reserve was assessed using the Child-Pugh classification (10). Laboratory variables, including platelet count (PLT) and total bilirubin (TBIL), were extracted from preoperative laboratory reports. Tumor-related variables, including tumor number, Barcelona Clinic Liver Cancer (BCLC) stage, and Edmondson-Steiner histological grade, were obtained from preoperative imaging records and postoperative pathological reports (3, 11). Child-Pugh class, BCLC stage, and Edmondson-Steiner grade were recorded as categorical variables to provide a complete description of liver function, tumor stage, and tumor differentiation status across the training and external validation cohorts.

Preoperative serological indices and tumor markers were retrospectively extracted from the electronic medical record system and laboratory information system. For each patient, the laboratory results closest to the preoperative CT examination and obtained before surgery were used for analysis. The extracted variables included platelet count, lymphocyte count, platelet-to-lymphocyte ratio (PLR), albumin, globulin, total bilirubin, alanine aminotransferase (ALT), aspartate aminotransferase (AST), alkaline phosphatase (ALP), alpha-fetoprotein (AFP), carcinoembryonic antigen (CEA), and carbohydrate antigen 199 (CA199). PLR was calculated as platelet count divided by lymphocyte count. Tumor marker positivity thresholds were predefined according to routine clinical laboratory cutoffs and previous literature: AFP ≥400 ng/mL, CEA ≥5 ng/mL, and CA199 ≥37 U/mL. The AFP threshold of 400 ng/mL was selected because this cutoff has been widely used in HCC studies and has been associated with aggressive tumor biology and increased MVI risk (12).

Preoperative CT features were retrospectively reviewed in both the training and external validation cohorts. Quantitative CT variables included maximum and minimum tumor diameters, CT attenuation values in the plain, arterial, portal venous, and delayed phases, enhancement degree, and enhancement rate. Enhancement degree was calculated as the difference between enhanced-phase and plain-scan CT attenuation, and enhancement rate was calculated as the ratio of enhanced-phase to plain-scan CT attenuation. Qualitative CT variables included tumor location, morphology, growth pattern, tumor margins, capsular interruption, arterial-phase ring enhancement, low-density ring sign, intratumoral hyperplastic vessels, satellite lesions, and the tumor-adjacent vessel relationship.

Tumor CT attenuation was measured three times at the largest solid tumor level, avoiding areas of hemorrhage, cystic degeneration, necrosis, and calcification, and the average value was used for analysis. An intact capsule was defined as capsular coverage of more than 90%. Arterial-phase ring enhancement was defined as crescent-shaped or sheet-like peritumoral enhancement in the arterial phase that decreased in the delayed phase. A low-density ring sign was defined as a hypodense ring surrounding the tumor in the portal venous phase. Intratumoral hyperplastic vessels were defined as persistent arterial vessel enhancement within the tumor during the arterial phase, with attenuation in subsequent phases. To minimize subjective bias, qualitative CT features were assessed using predefined imaging criteria. Where applicable, terminology and interpretation were referenced to the American College of Radiology Liver Imaging Reporting and Data System (LI-RADS) version 2018 (13). Study-specific imaging features, including intratumoral hyperplastic vessels and the tumor-adjacent vessel relationship, were defined before image review and assessed consistently by the two radiologists.

### Statistical analysis

This study utilized SPSS 25.0 and R software (version 4.5.0) for data processing. The normality of continuous variables was explicitly evaluated using the Kolmogorov-Smirnov test. Continuous variables adhering to a normal distribution (such as age, PLR, and albumin) were presented as mean ± standard deviation and compared using the independent samples t-test. Non-normally distributed continuous data (including total bilirubin, ALT, AST, and ALP) were expressed as medians (interquartile range) and analyzed using the non-parametric Mann–Whitney U-test. Categorical data were represented as frequencies (percentages), with group comparisons conducted using the chi-square test or Fisher’s exact test. Continuous variables with known clinical thresholds, such as AFP, were converted into pre-defined categorical variables (AFP ≥ 400 ng/mL) prior to modeling to handle potential non-linear relationships. Missing data were assessed during data extraction, and final cohorts consisted of complete cases without statistical imputation. Interobserver agreement for qualitative CT features was evaluated using Cohen’s kappa coefficient (κ).

To identify candidate predictors for MVI and reduce the risk of overfitting, a Least Absolute Shrinkage and Selection Operator (LASSO) regression model with 10-fold cross-validation was employed. Variables with non-zero coefficients at the optimal penalization parameter (minimum binomial deviance) were subsequently incorporated into a multivariable logistic regression model. The discriminative performance of the models was assessed using receiver operating characteristic (ROC) curves and the area under the curve (AUC). AUCs were compared using the DeLong test. The optimal cutoff value was determined by maximizing the Youden index in the training cohort and applied to the external validation cohort.

Model calibration was evaluated using calibration plots (with 1000 bootstrap resamples for internal validation) and the Hosmer–Lemeshow goodness-of-fit test. Decision curve analysis (DCA) was performed to quantify potential clinical net benefit. Furthermore, to evaluate whether the predictive performance of the final model was influenced by tumor burden, two sensitivity analyses related to tumor size were performed. First, maximum tumor diameter was forced into the final two-variable logistic regression model as an additional covariate to generate a tumor size-adjusted sensitivity model. The regression coefficients, odds ratios, and discriminatory performance of this extended model were compared with those of the original final model. Second, a subgroup analysis stratified by a clinically relevant tumor size cutoff (≤5 cm and >5 cm) was performed, and the AUCs between subgroups were compared using the DeLong test. These analyses were conducted to assess whether the associations of irregular tumor margins and AFP positivity with MVI, as well as the discrimination of the nomogram, remained stable across different tumor burden settings. Statistical significance was defined as a two-sided P < 0.05.

## Result

### Interobserver agreement

The interobserver agreement for the qualitative CT imaging features between the two independent radiologists was evaluated prior to model construction. The Cohen’s κ values for tumor margins, capsular interruption, and intratumoral hyperplastic vessels were 0.82 (95% CI: 0.74–0.90), 0.79 (95% CI: 0.68–0.90), and 0.85 (95% CI: 0.76–0.94), respectively. These results indicate substantial to almost perfect reproducibility in evaluating the imaging features across the expanded cohorts, ensuring the objectivity and reliability of the data utilized for subsequent modeling.

### Baseline clinicopathological characteristics

The baseline demographic, clinical, tumor-related, and serological characteristics stratified by MVI status are summarized in **Table 1**. In the training cohort, 187 patients were MVI-positive and 300 were MVI-negative. The two groups were generally comparable in demographic characteristics, viral hepatitis status, cirrhosis, liver functional reserve, platelet count, bilirubin level, tumor number, and BCLC stage, with no significant differences observed for these variables. However, Edmondson-Steiner grade III–IV was significantly more frequent in MVI-positive patients than in MVI-negative patients (P < 0.001), indicating a higher proportion of poorly differentiated tumors among patients with MVI.

**Table 1:** Baseline demographic, clinical, tumor-related, and serological characteristics of patients in the training and external validation cohorts.

Variables	Training cohort MVI+ (n = 187)	Training cohort MVI− (n = 300)	P value	External validation cohort MVI+ (n = 95)	External validation cohort MVI− (n = 161)	P value
Demographic and clinical features						
Age, years, mean ± SD	55.8 ± 10.5	56.8 ± 10.0	0.299	57.1 ± 9.8	56.5 ± 10.4	0.644
Male sex, n (%)	156 (83.4)	245 (81.7)	0.621	78 (82.1)	130 (80.7)	0.788
HBsAg positive, n (%)	160 (85.6)	250 (83.3)	0.512	82 (86.3)	136 (84.5)	0.689
Anti-HCV positive, n (%)	8 (4.3)	15 (5.0)	0.715	4 (4.2)	6 (3.7)	1.000
Cirrhosis, n (%)	148 (79.1)	226 (75.3)	0.333	76 (80.0)	125 (77.6)	0.657
Child-Pugh class, n (%)			0.403			1.000
Class A	175 (93.6)	286 (95.3)		91 (95.8)	154 (95.7)	
Class B	12 (6.4)	14 (4.7)		4 (4.2)	7 (4.3)	
PLT, ×10^9^/L, mean ± SD	165.4 ± 62.3	170.8 ± 58.6	0.342	161.2 ± 64.5	168.3 ± 60.1	0.384
TBIL, μmol/L, median (IQR)	14.2 (10.1–18.5)	13.8 (9.8–17.9)	0.285	14.5 (10.5–18.8)	13.9 (10.0–18.1)	0.412
Tumor-related characteristics						
Tumor number, n (%)			0.470			0.598
Solitary	159 (85.0)	262 (87.3)		81 (85.3)	141 (87.6)	
Multiple	28 (15.0)	38 (12.7)		14 (14.7)	20 (12.4)	
BCLC stage, n (%)			0.390			0.508
Stage A	165 (88.2)	272 (90.7)		85 (89.5)	148 (91.9)	
Stage B	22 (11.8)	28 (9.3)		10 (10.5)	13 (8.1)	
Edmondson-Steiner grade, n (%)			<0.001			0.013
Grade I–II	108 (57.8)	227 (75.7)		56 (58.9)	119 (73.9)	
Grade III–IV	79 (42.2)	73 (24.3)		39 (41.1)	42 (26.1)	
Serological indices						
PLR, mean ± SD	144.51 ± 86.56	138.71 ± 80.00	0.452	140.47 ± 95.22	141.83 ± 94.97	0.905
Albumin, g/L, mean ± SD	39.97 ± 5.51	39.49 ± 5.67	0.356	40.23 ± 5.05	41.17 ± 4.20	0.128
Globulin, g/L, mean ± SD	30.38 ± 7.74	29.35 ± 7.07	0.141	30.11 ± 5.83	30.60 ± 4.72	0.488
ALT, U/L, median (IQR)	36.5 (24.0–55.0)	48.0 (29.0–89.0)	0.021	39.0 (25.0–65.0)	43.0 (27.0–78.0)	0.125
AST, U/L, median (IQR)	40.0 (26.0–58.0)	46.0 (28.0–72.0)	0.085	44.0 (30.0–65.0)	50.0 (32.0–80.0)	0.061
ALP, U/L, median (IQR)	95.0 (72.0–128.0)	92.0 (68.0–125.0)	0.671	96.0 (74.0–135.0)	105.0 (78.0–155.0)	0.076
Tumor markers						
AFP, n (%)			<0.001			0.002
Negative (<400 ng/mL)	37 (19.8)	145 (48.3)		19 (20.0)	63 (39.1)	
Positive (≥400 ng/mL)	150 (80.2)	155 (51.7)		76 (80.0)	98 (60.9)	
CEA, n (%)			0.893			0.054
Negative (<5 ng/mL)	153 (81.8)	244 (81.3)		90 (94.7)	140 (87.0)	
Positive (≥5 ng/mL)	34 (18.2)	56 (18.7)		5 (5.3)	21 (13.0)	
CA199, n (%)			0.061			0.147
Negative (<37 U/mL)	180 (96.3)	276 (92.0)		91 (95.8)	145 (90.1)	
Positive (≥37 U/mL)	7 (3.7)	24 (8.0)		4 (4.2)	16 (9.9)	

Continuous variables conforming to a normal distribution are presented as mean ± standard deviation and were compared using the independent samples t-test. Non-normally distributed variables, including TBIL, ALT, AST, and ALP, are presented as median (interquartile range) and were compared using the Mann-Whitney U test. Categorical variables are presented as number (percentage) and were compared using the chi-square test or Fisher’s exact test. MVI, microvascular invasion; HBsAg, hepatitis B surface antigen; HCV, hepatitis C virus; PLT, platelet; TBIL, total bilirubin; BCLC, Barcelona Clinic Liver Cancer; PLR, platelet/lymphocyte ratio; ALT, alanine aminotransferase; AST, aspartate aminotransferase; ALP, alkaline phosphatase; AFP, alpha-fetoprotein; CEA, carcinoembryonic antigen; CA199, carbohydrate antigen 199; SD, standard deviation; IQR, interquartile range.

Regarding serological indices and tumor markers, AFP positivity (≥400 ng/mL) was markedly more frequent in the MVI-positive group than in the MVI-negative group in the training cohort (P < 0.001). ALT also differed between the two groups, P = 0.021], whereas PLR, albumin, globulin, AST, ALP, CEA positivity, and CA199 positivity showed no significant between-group differences.

Similar baseline patterns were observed in the external validation cohort. Most demographic, liver disease-related, and tumor burden-related variables were comparable between MVI-positive and MVI-negative patients. Consistent with the training cohort, Edmondson-Steiner grade III–IV was more common in MVI-positive patients (P = 0.013), and AFP positivity was also significantly more frequent in the MVI-positive group (P = 0.002). These findings support the reproducibility of the main clinicopathological trends across independent cohorts.

The overall comparison between the training and external validation cohorts is presented in **Supplementary Table 1**. Most baseline variables were well balanced between the two cohorts, including age, sex, viral hepatitis status, cirrhosis, Child-Pugh class, tumor number, BCLC stage, Edmondson-Steiner grade, and AFP positivity. Albumin level was slightly higher in the external validation cohort (P = 0.003), whereas CEA positivity was less frequent (P = 0.003). These differences were reported transparently, but neither variable was retained in the final predictive model.

### Baseline preoperative CT imaging characteristics

A comparative analysis of preoperative CT qualitative and quantitative features between MVI-positive and MVI-negative patients is presented in **Table 2**. In the training cohort, MVI-positive tumors more frequently showed irregular margins (P < 0.001), capsular interruption (P < 0.001), and intratumoral hyperplastic vessels (P < 0.001) than MVI-negative tumors. These three imaging features were subsequently retained during LASSO-based feature selection. Other CT features, including tumor morphology (P = 0.022), satellite lesions (P = 0.041), and the tumor-adjacent vessel relationship (P = 0.021), also differed between groups in the univariable comparison.

**Table 2:** Analysis of preoperative CT qualitative and quantitative features in the training and external validation cohorts.

Variables	Training cohort MVI+ (n = 187)	Training cohort MVI− (n = 300)	P value	External validation cohort MVI+ (n = 95)	External validation cohort MVI− (n = 161)	P value
Qualitative features, n (%)						
Location			0.654			0.635
Right lobe	149 (79.7)	244 (81.3)		72 (75.8)	116 (72.0)	
Left lobe	38 (20.3)	56 (18.7)		21 (22.1)	43 (26.7)	
Caudate lobe	0 (0.0)	0 (0.0)		2 (2.1)	2 (1.2)	
Morphology			0.022			0.081
Irregular	137 (73.3)	183 (61.0)		69 (72.6)	100 (62.1)	
Circular	4 (2.1)	10 (3.3)		0 (0.0)	0 (0.0)	
Orbicular	46 (24.6)	107 (35.7)		26 (27.4)	61 (37.9)	
Growth pattern			0.903			0.072
Intrahepatic	146 (78.1)	233 (77.7)		73 (76.8)	138 (85.7)	
Extrahepatic	41 (21.9)	67 (22.3)		22 (23.2)	23 (14.3)	
Margins			<0.001			<0.001
Regular	34 (18.2)	162 (54.0)		17 (17.9)	110 (68.3)	
Irregular	153 (81.8)	138 (46.0)		78 (82.1)	51 (31.7)	
Capsular interruption			<0.001			<0.001
Positive	82 (43.9)	42 (14.0)		52 (54.7)	25 (15.5)	
Negative	105 (56.1)	258 (86.0)		43 (45.3)	136 (84.5)	
Ring enhancement			0.071			0.884
Positive	40 (21.4)	45 (15.0)		24 (25.3)	42 (26.1)	
Negative	147 (78.6)	255 (85.0)		71 (74.7)	119 (73.9)	
Low-density ring sign			0.468			0.730
Positive	10 (5.3)	21 (7.0)		12 (12.6)	18 (11.2)	
Negative	177 (94.7)	279 (93.0)		83 (87.4)	143 (88.8)	
Intratumoral hyperplastic vessels			<0.001			<0.001
Positive	150 (80.2)	173 (57.7)		86 (90.5)	85 (52.8)	
Negative	37 (19.8)	127 (42.3)		9 (9.5)	76 (47.2)	
Satellite lesion			0.041			0.865
Positive	13 (7.0)	9 (3.0)		14 (14.7)	25 (15.5)	
Negative	174 (93.0)	291 (97.0)		81 (85.3)	136 (84.5)	
Tumor-adjacent vessel relationship			0.021			0.435
Close	135 (72.2)	186 (62.0)		52 (54.7)	80 (49.7)	
Distant	52 (27.8)	114 (38.0)		43 (45.3)	81 (50.3)	
Quantitative features, mean ± SD						
Tumor size, cm						
Maximum diameter	6.55 ± 3.83	5.60 ± 3.27	0.005	6.49 ± 3.23	5.90 ± 3.31	0.165
Minimum diameter	5.30 ± 3.10	4.48 ± 2.36	0.002	5.40 ± 2.61	4.95 ± 2.55	0.181
Tumor CT values, HU						
Plain scan	45.34 ± 7.54	46.05 ± 7.54	0.315	45.22 ± 6.37	46.03 ± 6.98	0.342
Arterial phase	102.25 ± 54.55	99.64 ± 44.32	0.582	92.06 ± 49.64	86.50 ± 33.72	0.335
Portal venous phase	100.75 ± 32.24	100.58 ± 23.69	0.951	99.55 ± 25.46	101.45 ± 22.44	0.548
Delayed phase	86.18 ± 17.93	87.01 ± 16.83	0.612	84.41 ± 16.10	85.89 ± 15.32	0.470
Enhancement degree, HU						
Arterial phase	56.94 ± 52.77	53.59 ± 43.59	0.468	47.47 ± 47.85	40.47 ± 34.75	0.215
Portal venous phase	55.12 ± 29.84	54.53 ± 21.00	0.812	54.38 ± 24.14	55.42 ± 20.53	0.725
Delayed phase	41.07 ± 15.35	40.96 ± 14.65	0.940	39.23 ± 13.62	39.87 ± 13.69	0.718
Enhancement rate						
Arterial phase	1.27 ± 1.11	1.20 ± 1.02	0.495	1.07 ± 1.05	0.94 ± 0.93	0.316
Portal venous phase	1.23 ± 0.62	1.21 ± 0.49	0.641	1.22 ± 0.54	1.23 ± 0.52	0.930
Delayed phase	0.92 ± 0.36	0.91 ± 0.37	0.782	0.88 ± 0.31	0.89 ± 0.36	0.895

Qualitative variables are presented as number (percentage) and were compared using the chi-square test or Fisher exact test, as appropriate. Quantitative variables are presented as mean ± standard deviation and were compared using the independent samples t-test. P values for variables with more than two categories were calculated across all categories. MVI, microvascular invasion; HU, Hounsfield unit; SD, standard deviation.

Regarding quantitative CT features, MVI-positive patients had larger tumors than MVI-negative patients in the training cohort, with significant differences in both maximum tumor diameter (P = 0.005) and minimum tumor diameter (P = 0.002). In contrast, CT attenuation values and enhancement-related quantitative parameters showed no consistent significant differences between MVI-positive and MVI-negative patients in the training cohort.

In the external validation cohort, the main imaging patterns were generally consistent with those observed in the training cohort. Irregular margins (P < 0.001), capsular interruption (P < 0.001), and intratumoral hyperplastic vessels (P < 0.001) remained significantly more frequent in MVI-positive patients. Tumor size was numerically larger in MVI-positive patients, but the differences in maximum and minimum tumor diameters did not reach statistical significance (P = 0.165 and P = 0.181, respectively).

### Feature selection via LASSO regression

To overcome the limitations of traditional univariable screening and to rigorously prevent model overfitting, a LASSO regression was utilized for high-dimensional feature selection. All preoperative clinical serological indices and CT imaging characteristics (comprising over 20 variables) from the training cohort (n=487) were incorporated into the LASSO algorithm.

**Figure 2:**
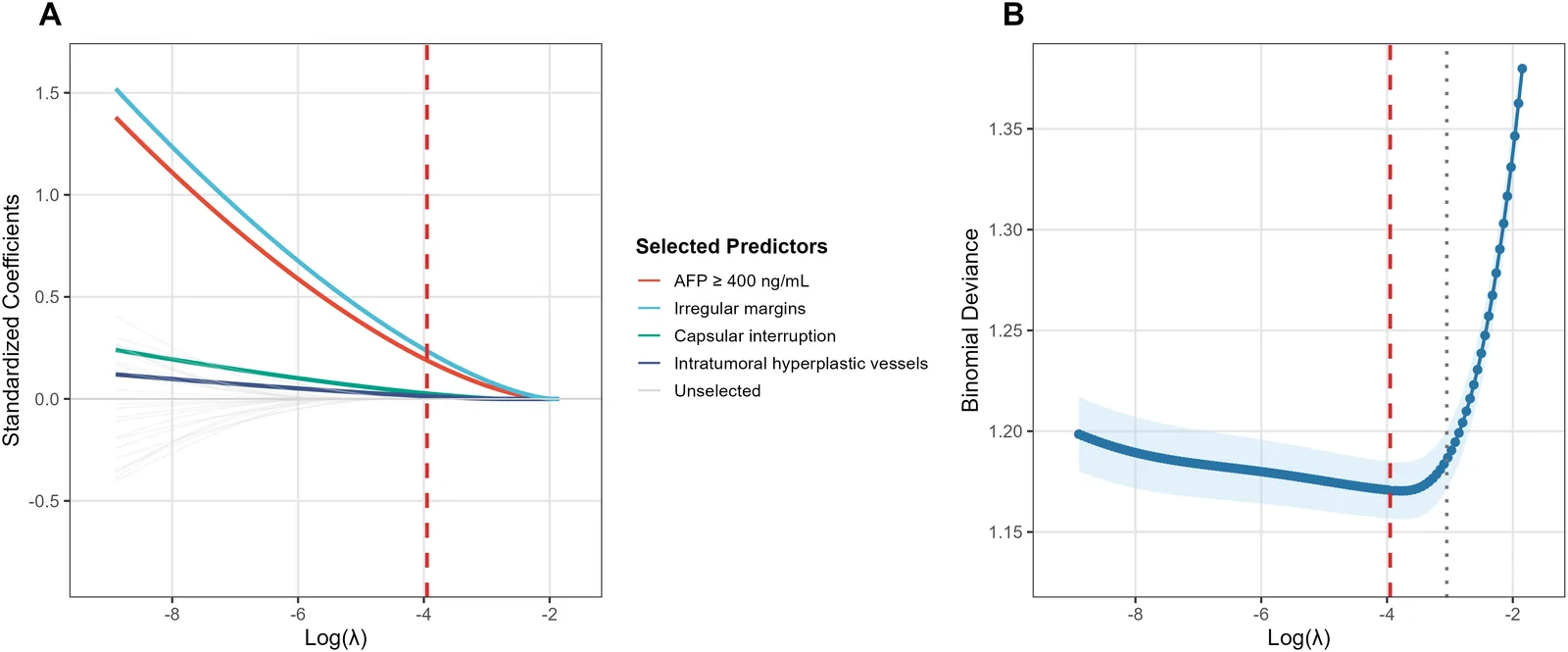
Feature selection using the Least Absolute Shrinkage and Selection Operator (LASSO) binary logistic regression model. **(A)** LASSO coefficient profile plot of the candidate preoperative clinical and CT imaging features. A red dashed vertical line indicates the optimal penalty parameter selected by 10-fold cross-validation (λmin), at which features with non-zero coefficients were identified. **(B)** Ten-fold cross-validation curve for tuning parameter selection in the LASSO model. Binomial deviance is plotted against log(λ). The red dashed vertical line indicates λmin (minimum criteria), and the gray dotted vertical line indicates λ1se (1 standard error of the minimum criteria).

A 10-fold cross-validation was strictly employed to calculate the optimal penalization parameter (λ). As illustrated in the LASSO coefficient profile plot (**Figure 2A**), the coefficients of most candidate variables were gradually shrunk toward zero as the penalty increased, reducing the influence of redundant or weakly informative predictors. The optimal λ value was determined based on the minimum binomial deviance criteria (**Figure 2B**). At λmin, four variables retained non-zero coefficients and were selected for further analysis: alpha-fetoprotein positivity (AFP ≥ 400 ng/mL), irregular tumor margins, capsular interruption, and intratumoral hyperplastic vessels. These selected variables were subsequently incorporated into the multivariable logistic regression analysis to construct the final predictive model.

### Multivariable logistic regression analysis and nomogram construction

To further identify the independent predictors of MVI and construct a quantitative prediction model, the four LASSO-selected variables, including AFP positivity, irregular tumor margins, capsular interruption, and intratumoral hyperplastic vessels, were entered into a multivariable logistic regression model in the expanded training cohort.

The training cohort contained 187 MVI-positive events. With four predictors entered into the adjusted model, the events-per-variable ratio was 46.8, exceeding the commonly recommended threshold and thereby reducing the risk of model overfitting.

Among the four LASSO-selected variables, irregular tumor margins and AFP positivity remained independently associated with MVI after adjustment. Specifically, irregular tumor margins were strongly associated with increased odds of MVI (adjusted OR = 5.275, 95% CI: 3.165–8.791, P < 0.001), as was AFP positivity (≥ 400 ng/mL) (adjusted OR = 3.297, 95% CI: 1.983–5.481, P < 0.001). In contrast, capsular interruption and intratumoral hyperplastic vessels did not remain statistically significant after adjustment (**Table 3**). Therefore, irregular tumor margins and AFP positivity were incorporated into the final nomogram for individualized prediction of MVI risk in HCC patients. For reproducibility, the final two-variable logistic model was expressed as follows: logit (PMVI) = −2.364 + 1.663 × irregular margins + 1.193 × AFP positivity, where irregular margins and AFP positivity were coded as 1 when present and 0 when absent.

**Table 3:** Multivariable logistic regression analysis of independent risk factors for MVI in the expanded training cohort.

Variables	Regression coefficient (β)	Standard error (SE)	Wald χ²	P value	Odds ratio (OR)	95% CI of OR
Irregular margins	1.663	0.261	40.612	< 0.001	5.275	3.165–8.791
AFP (≥ 400 ng/mL)	1.193	0.26	21.053	< 0.001	3.297	1.983–5.481
Capsular interruption	0.329	0.246	1.789	0.181	1.389	0.858–2.249
Intratumoral hyperplastic vessels	0.404	0.297	1.851	0.174	1.497	0.836–2.680

Variables incorporated into this multivariable model were pre-selected by the LASSO regression algorithm. The final nomogram was constructed using the independently significant predictors, including irregular tumor margins and AFP positivity. For the final two-variable model, irregular margins and AFP positivity were coded as 1 when present and 0 when absent. AFP, alpha-fetoprotein; OR, odds ratio; CI, confidence interval.

**Figure 3:**
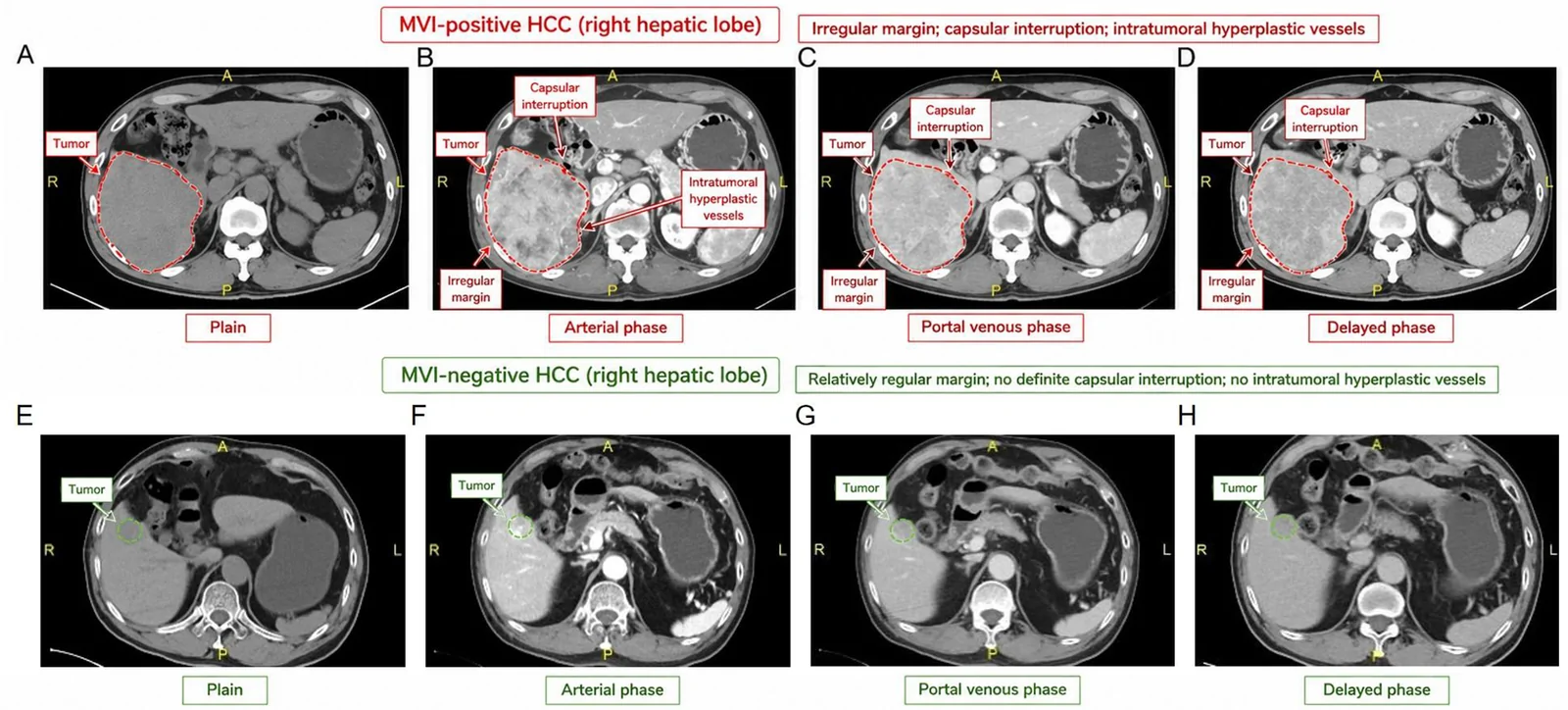
Representative dynamic contrast-enhanced CT images contrasting typical preoperative imaging features between MVI-positive and MVI-negative hepatocellular carcinoma (HCC) patients. **(A–D)** MVI-positive HCC (alpha-fetoprotein [AFP] ≥ 400 ng/mL) in the right hepatic lobe. **(A)** The plain scan shows a heterogeneous low-density mass. **(B–D)** Dynamic enhancement phases highlight classical high-risk imaging features: an irregular tumor margin (red dashed outline), discernible capsular interruption, and the presence of intratumoral hyperplastic vessels (red arrows). These features were more frequently observed in MVI-positive tumors and were selected during the LASSO-based feature selection process. **(E–H)** MVI-negative HCC (AFP < 400 ng/mL) in the right hepatic lobe. **(E)** The plain scan reveals a more homogeneous low-density mass. **(F–H)** In contrast to the MVI-positive case, this tumor demonstrates relatively regular margins (green dashed outline), an intact capsule without definite interruption, and an absence of evident intratumoral hyperplastic vessels. Both cases illustrate the characteristic “wash-in and wash-out” enhancement pattern typical of HCC. HCC, hepatocellular carcinoma; MVI, microvascular invasion.

Representative preoperative CT images illustrating the characteristic radiological signs of MVI-positive and MVI-negative HCC patients are presented in **Figure 3**.

### Model discrimination and optimal cutoff selection

To evaluate the discrimination performance of the predictive models and establish standardized clinical thresholds, ROC curve analysis was conducted. Three distinct prediction models were compared: the clinical model (incorporating only AFP), the imaging model (incorporating only tumor margins), and the combined model (incorporating both AFP and tumor margins).

To strictly address the selection of diagnostic thresholds, the optimal cutoff value for the predicted probability was determined by maximizing the Youden index (sensitivity + specificity - 1) derived from the ROC curve of the combined model in the training cohort. This pre-specified absolute threshold was subsequently applied to both the training and external validation cohorts to systematically calculate the accuracy, sensitivity, and specificity.

**Figure 4:**
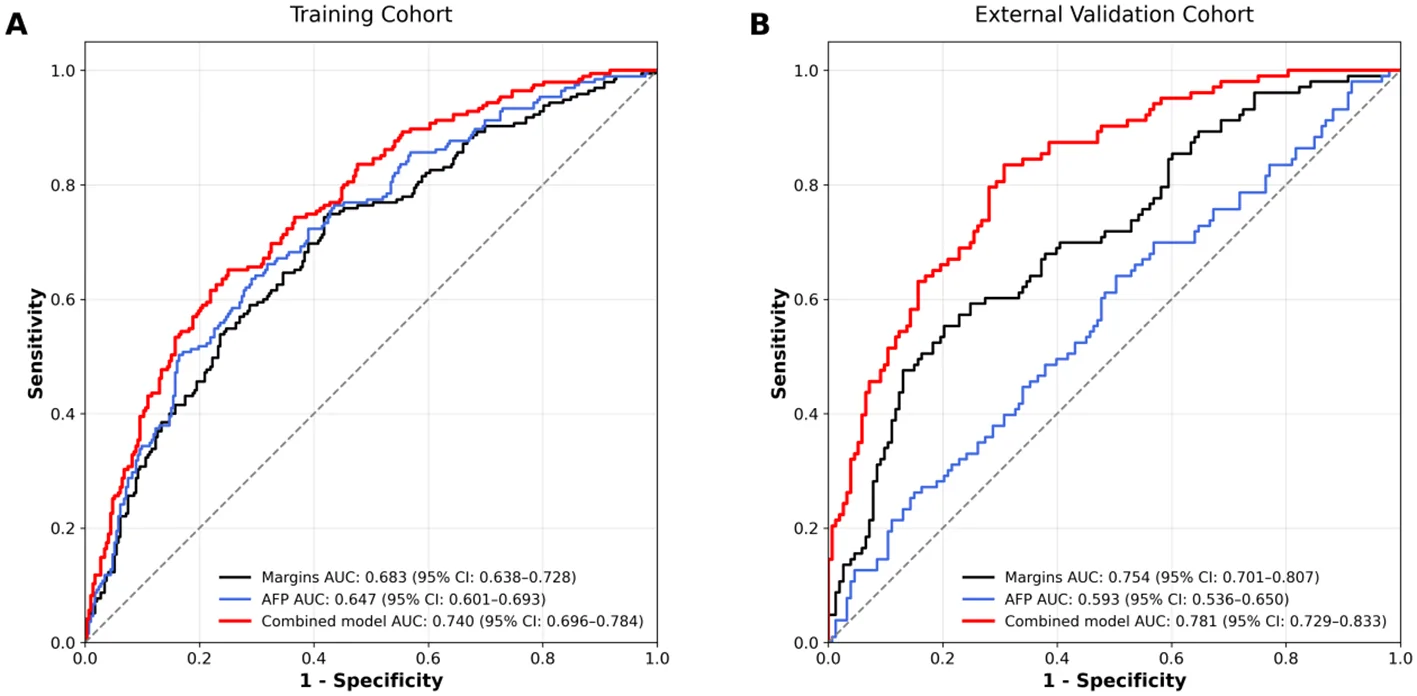
Receiver operating characteristic (ROC) curves evaluating the diagnostic performance of the prediction models for microvascular invasion. **(A)** Comparison of the ROC curves for the combined model, tumor margins alone, and alpha-fetoprotein (AFP) alone in the training cohort (n=487). **(B)** Validation of the ROC curves for the respective models in the independent external validation cohort (n=256). The red solid line represents the combined predictive model, the black solid line represents the imaging model based on irregular tumor margins, and the blue solid line represents the clinical model based on AFP positivity. The diagonal dashed gray line indicates the reference line for a random guess. AUC, area under the curve; CI, confidence interval; AFP, alpha-fetoprotein.

**Figure 5:**
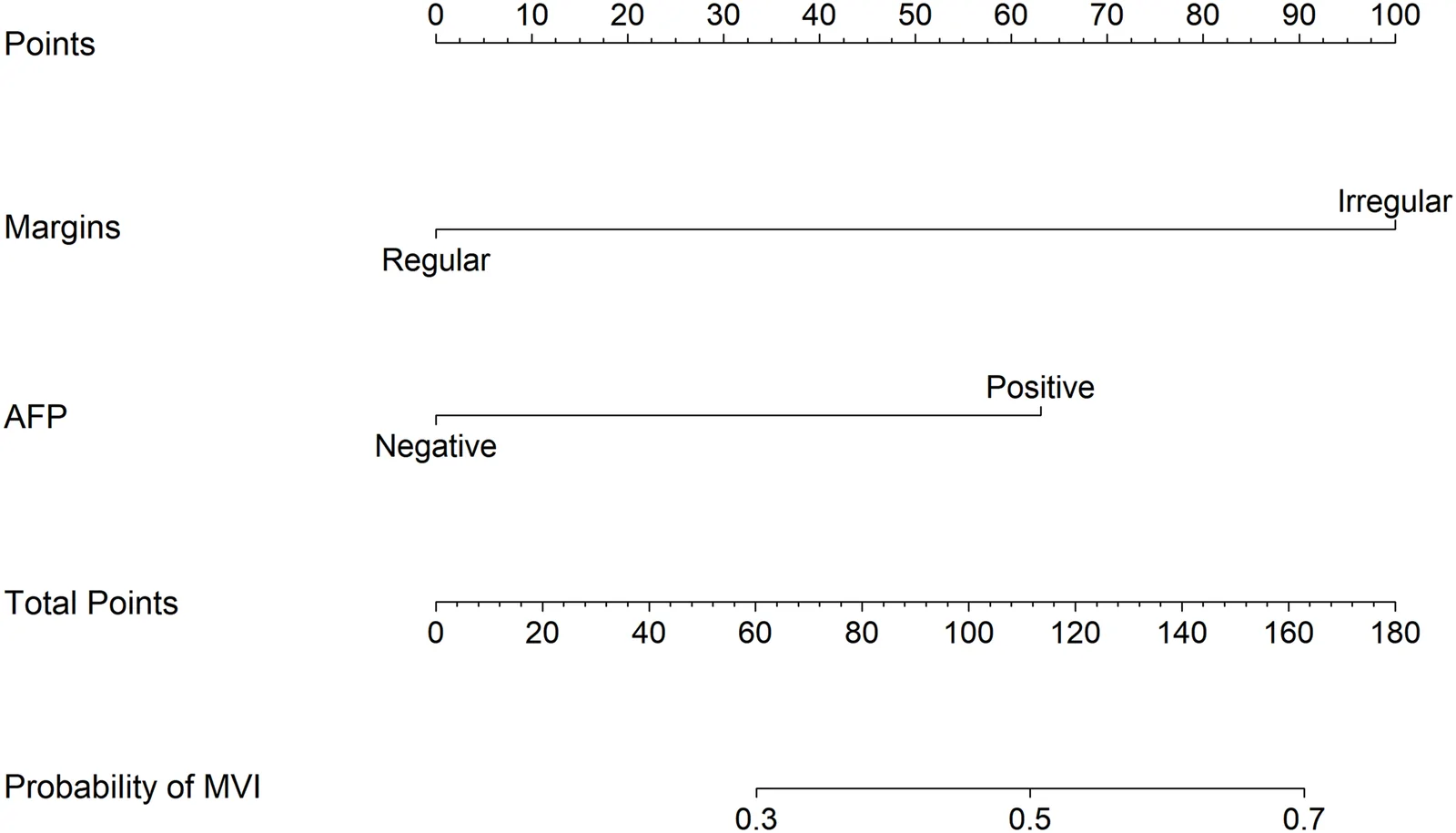
Nomogram for exploratory prediction of microvascular invasion (MVI) risk in patients with hepatocellular carcinoma. The nomogram was developed using the independently significant predictors identified in the training cohort, including irregular tumor margins and alpha-fetoprotein (AFP) positivity. The final two-variable logistic model was defined as logit (PMVI) = −2.364 + 1.663 × irregular margins + 1.193 × AFP positivity, with both predictors coded as 1 when present and 0 when absent. To use the nomogram, locate the patient’s status for each predictor on its corresponding axis, assign points, sum the total points, and project the value to the probability axis to estimate the preoperative probability of MVI. AFP, alpha-fetoprotein; MVI, microvascular invasion.

The ROC curve analysis (**Figure 4**) showed that the combined model achieved better discrimination than the individual clinical and imaging models. In the training cohort, the combined model achieved an AUC of 0.740 (95% CI: 0.696–0.784), with an accuracy of 70.5%, a sensitivity of 67.3%, and a specificity of 72.6%. DeLong tests showed that the combined model had a significantly higher AUC than both the AFP model and the margins model in the training cohort (both P < 0.05). In the independent external validation cohort, the combined model showed acceptable diagnostic performance, yielding an AUC of 0.781 (95% CI: 0.729–0.833), an accuracy of 73.3%, a sensitivity of 81.8%, and a specificity of 68.4%. The combined model significantly outperformed the AFP model in the external validation cohort (DeLong P < 0.001), whereas its AUC was numerically higher than that of the margins model without reaching statistical significance (DeLong P = 0.214). The detailed diagnostic performance metrics and AUC comparison results are summarized in **Table 4**. Based on the observed predictive performance of the combined predictors, a nomogram for estimating the probability of MVI was constructed (**Figure 5**).

**Table 4:** Diagnostic value of individual and combined models for detecting MVI using ROC curve analysis.

Cohort	Prediction model	AUC	95% CI for AUC	Accuracy	Sensitivity	Specificity	DeLong P vs combined model
Training cohort	Margins	0.683	0.638–0.728	61.1%	81.8%	54.8%	0.018
	AFP	0.647	0.601–0.693	65.0%	81.8%	47.6%	<0.001
	Combined model	0.740	0.696–0.784	70.5%	67.3%	72.6%	Reference
External validation cohort	Margins	0.754	0.701–0.807	73.3%	81.8%	68.4%	0.214
	AFP	0.593	0.536–0.650	53.3%	81.2%	36.8%	<0.001
	Combined model	0.781	0.729–0.833	73.3%	81.8%	68.4%	Reference

The optimal cutoff value was determined based on the maximum Youden index in the training cohort and consistently applied to the external validation cohort. AUC, area under the curve; CI, confidence interval; AFP, alpha-fetoprotein.

### Model calibration and clinical utility assessment

**Figure 6:**
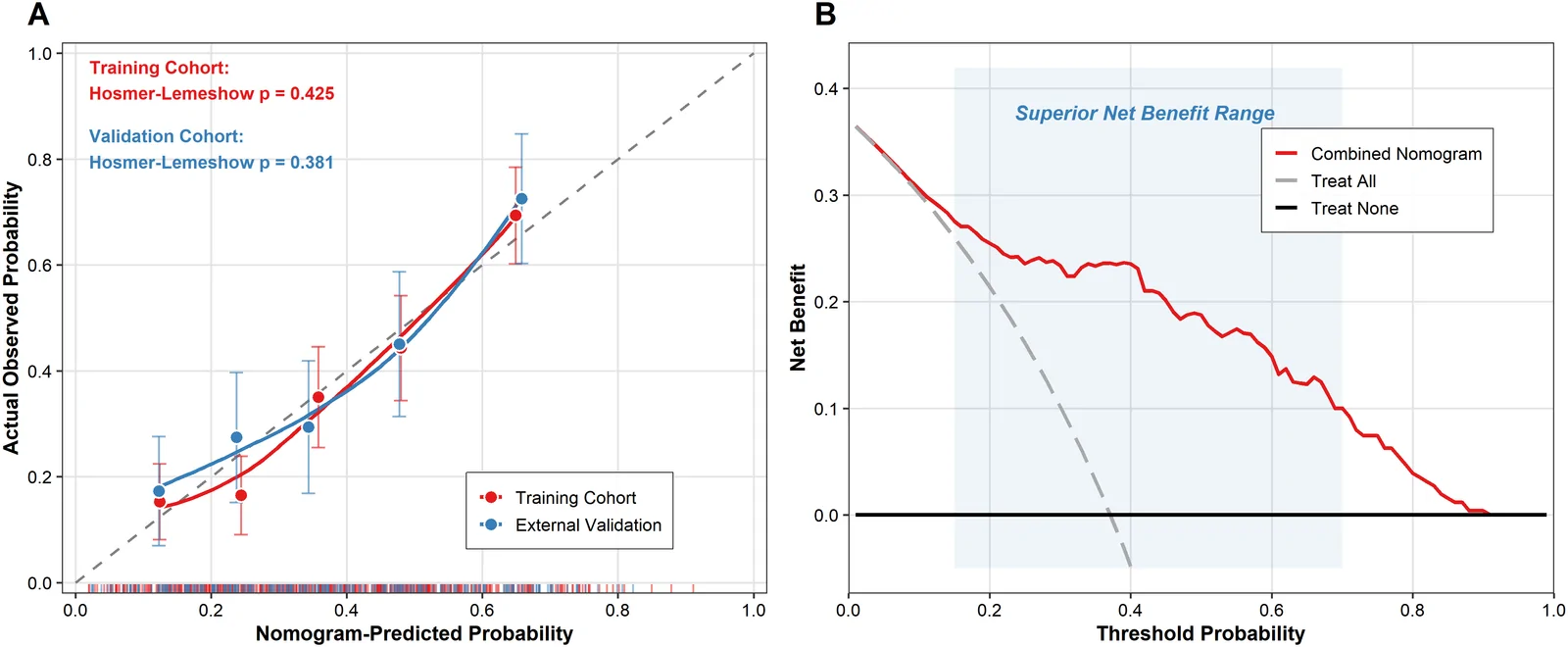
Calibration and clinical utility of the combined predictive nomogram. **(A)** Calibration plots for predicting MVI probability in the training cohort (internal validation via 1000 bootstrap resamples) and the external validation cohort. The x-axis represents the nomogram-predicted probability, and the y-axis represents the actual observed rate of MVI. The diagonal dashed line indicates a perfect prediction, while the solid lines represent the performance of the nomogram. **(B)** Decision curve analysis (DCA) of the combined model in the external validation cohort. The y-axis measures the net benefit. The colored solid line represents the combined predictive model, the descending solid gray line assumes all patients have MVI (treat-all strategy), and the horizontal solid black line assumes no patients have MVI (treat-none strategy). The nomogram showed a higher net benefit than the treat-all and treat-none strategies across a clinically relevant range of threshold probabilities, suggesting potential clinical utility that requires further prospective validation.

To assess the goodness-of-fit and potential clinical utility of the combined prediction model, calibration plots and DCA were performed (**Figure 6**).

The calibration curves were generated to visualize the agreement between the nomogram-predicted probabilities of MVI and the observed frequencies. In the training cohort, internal validation using 1000 bootstrap resamples showed good agreement between predicted and observed probabilities, with the calibration curve generally following the ideal 45-degree diagonal line. Similar calibration performance was observed in the independent external validation cohort (**Figure 6A**). The Hosmer–Lemeshow goodness-of-fit test yielded non-significant results in both the training (P = 0.425) and external validation (P = 0.381) cohorts, suggesting no statistically significant deviation between predicted risks and observed outcomes. Furthermore, quantitative calibration metrics demonstrated excellent agreement. In the training cohort, the calibration intercept was 0.015 (95% CI: -0.124 to 0.154) and the calibration slope was 0.982 (95% CI: 0.865 to 1.099). In the external validation cohort, the calibration intercept and slope were -0.042 (95% CI: -0.218 to 0.134) and 0.945 (95% CI: 0.792 to 1.098), respectively, confirming the robustness and reliability of the nomogram’s predicted probabilities.

Beyond statistical discrimination and calibration, the potential clinical utility of the nomogram was evaluated using DCA. As illustrated in **Figure 6B**, the decision curve indicated that the combined nomogram provided a higher net benefit than the “treat-all” and “treat-none” strategies across a clinically relevant range of threshold probabilities. Collectively, these analyses suggest that the proposed combined model may have potential value for preoperative MVI risk stratification, but further prospective and multicenter validation is needed before it can be used to guide treatment decisions.

### Subgroup analysis based on tumor size

Because tumor size was larger in MVI-positive patients in the training cohort, additional sensitivity analyses were performed to evaluate whether the final model was materially influenced by tumor burden. First, maximum tumor diameter was forced into the final two-variable logistic regression model. In this tumor size-adjusted sensitivity model, irregular tumor margins and AFP positivity remained independently associated with MVI, with effect estimates similar to those in the original final model. The addition of maximum tumor diameter produced only a small numerical increase in discrimination compared with the final model (AUC, 0.748 vs. 0.740; DeLong P = 0.218), indicating limited incremental predictive value after accounting for irregular margins and AFP positivity (**Supplementary Table 2**).

Second, subgroup analysis was performed according to a clinically relevant tumor size cutoff of 5 cm. As shown in **Table 5**, the combined model achieved comparable discrimination in patients with maximum tumor diameter ≤5 cm and >5 cm in the training cohort, with AUCs of 0.725 (95% CI: 0.658–0.792) and 0.751 (95% CI: 0.690–0.812), respectively (DeLong P = 0.543). Similar findings were observed in the external validation cohort, with AUCs of 0.768 (95% CI: 0.692–0.844) and 0.795 (95% CI: 0.718–0.872) in the ≤5 cm and >5 cm subgroups, respectively (DeLong P = 0.612). These results suggest that the discriminatory performance of the final model was generally consistent across different tumor size categories.

**Table 5:** Subgroup analysis of discriminatory performance stratified by tumor size.

Cohort	Tumor size subgroup	n	AUC (95% CI)	P value*
Training Cohort	≤ 5 cm	231	0.725 (0.658–0.792)	0.543
	> 5 cm	256	0.751 (0.690–0.812)	
External Validation Cohort	≤ 5 cm	125	0.768 (0.692–0.844)	0.612
	> 5 cm	131	0.795 (0.718–0.872)	

*The P value was calculated using the DeLong test to compare the area under the curve (AUC) between the ≤ 5 cm and > 5 cm subgroups within the same cohort. CI, confidence interval.

## Discussion

HCC remains a major malignancy worldwide and is associated with substantial recurrence and mortality after curative-intent treatment (14). MVI is a histopathological manifestation of aggressive tumor biology and is usually defined as microscopic tumor emboli within vascular spaces lined by endothelial cells. Although MVI is typically confirmed only after surgical resection, it has important prognostic implications because it is strongly associated with postoperative recurrence and reduced survival (15). Therefore, preoperative MVI risk estimation has become an important research direction in HCC, especially for patients who are being considered for potentially curative treatment.

Numerous studies have developed preoperative MVI prediction models using conventional CT findings, MRI features, radiomics, deep learning, contrast-enhanced ultrasound, and serological markers (15–22). Several high-dimensional radiomics or deep-learning models have reported favorable discrimination by extracting quantitative features from preoperative imaging, but these approaches often require tumor segmentation, image normalization, feature extraction, software support, and local technical validation before routine use (16–18). Conventional CT-based models are more feasible in routine radiological practice, but their performance depends on reproducible visual interpretation and external validation (19). Against this background, the present study should not be positioned as a conceptually novel replacement for previously reported MVI prediction models. Its main value is the development and external validation of a simple, interpretable, and reproducible nomogram based on routinely available CT margin assessment and AFP positivity. This model provides a pragmatic risk-stratification approach rather than a comprehensive substitute for radiomics, MRI-based, or biomarker-expanded models.

AFP was retained in the final model, which is biologically and clinically plausible. AFP is a widely used HCC biomarker and has been associated with tumor burden, aggressive tumor behavior, recurrence risk, and long-term prognosis (23, 24). However, AFP alone has limited predictive ability because a proportion of HCC patients with aggressive pathological features may have low AFP levels, while elevated AFP may also reflect tumor burden or underlying liver disease activity rather than MVI specifically. Other serum markers, such as alpha-fetoprotein-L3 (AFP-L3) and prothrombin induced by vitamin K absence-II (PIVKA-II), also known as des-gamma-carboxy prothrombin (DCP), have been reported to reflect aggressive tumor biology and may provide additional information for HCC risk stratification (25, 26). In particular, PIVKA-II levels have been associated with MVI and tumor cell proliferation in HBV-related HCC (26). These findings support the need to compare the present AFP-based model with biomarker-expanded models in future prospective cohorts. In addition, because AFP expression and tumor imaging phenotypes may differ across HCC etiologies, the AFP-based component of this model should be interpreted within the etiological context of the present cohorts. The model was developed and validated predominantly in patients with HBV-related HCC, whereas its performance in HCV-related, alcohol-related, metabolic dysfunction-associated steatotic liver disease (MASLD)-associated, or metabolic dysfunction-associated steatohepatitis (MASH)-associated HCC remains to be evaluated. Caution is therefore warranted when applying this nomogram to populations in which HBV infection is not the predominant risk factor.

Among the CT features evaluated in this study, irregular tumor margins, capsular interruption, and intratumoral hyperplastic vessels differed between MVI-positive and MVI-negative tumors and were retained during LASSO-based feature selection. These features are consistent with the concept that MVI-positive HCC may show imaging phenotypes related to infiltrative growth, capsular disruption, tumor vascularity, and aggressive biological behavior (19, 27). However, the multivariable logistic regression model clarified that only irregular tumor margins and AFP positivity remained independently associated with MVI after adjustment. This distinction is central to the interpretation of the final model. Capsular interruption and intratumoral hyperplastic vessels should be interpreted as MVI-related imaging characteristics and LASSO-selected candidate features, but not as independent predictors retained in the final nomogram.

Irregular tumor margins were the strongest independent imaging predictor in the final model. This result is clinically plausible because irregular margins may reflect infiltrative tumor growth at the tumor-liver interface and microscopic extension beyond the apparent tumor boundary (27). Compared with quantitative enhancement values, tumor margin assessment is relatively intuitive and can be obtained from routine multiphasic CT interpretation. Nevertheless, qualitative imaging features may be affected by reader experience, CT protocol, and image quality. To address this issue, the present study added an interobserver agreement analysis for key imaging features. The substantial to almost perfect κ values support the reliability of these assessments in the available cohorts, although further standardization across centers remains necessary.

A major methodological revision of the present study was the use of LASSO regression for candidate predictor selection. This approach was applied to reduce reliance on simple univariable screening and to mitigate overfitting when multiple preoperative clinical and imaging variables were considered. LASSO selected four variables with non-zero coefficients: AFP positivity, irregular tumor margins, capsular interruption, and intratumoral hyperplastic vessels. These variables were subsequently entered into multivariable logistic regression, and the final nomogram was restricted to irregular tumor margins and AFP positivity because only these two variables remained independently associated with MVI after adjustment. This modeling process improves transparency by separating LASSO-based feature selection from final independent predictor identification. In addition, the final two-variable regression equation was provided to improve reproducibility and to allow external testing in other cohorts.

The discriminatory performance of the final model should be interpreted cautiously. The combined model achieved an AUC of 0.740 in the training cohort and 0.781 in the independent external validation cohort, indicating fair-to-acceptable discrimination rather than strong predictive performance. DeLong testing showed that the combined model had better discrimination than AFP alone in both cohorts, while the improvement over tumor margin alone in the external validation cohort was numerical but not statistically significant. These findings suggest that AFP provides some complementary information to imaging-based assessment, but the incremental predictive gain of the two-variable model is modest. Therefore, the model should not be presented as superior to existing radiomics, MRI-based, or multi-biomarker models. Its strength is mainly its simplicity, interpretability, and feasibility in routine clinical settings where complex image-processing pipelines are unavailable.

The present study also added calibration assessment and DCA, because AUC alone is insufficient for evaluating a clinical prediction model. Calibration plots and Hosmer-Lemeshow tests suggested acceptable agreement between predicted and observed MVI risks in the available cohorts. DCA suggested that the combined model may provide net benefit across a clinically relevant range of threshold probabilities compared with treat-all and treat-none strategies. However, DCA does not prove that model-guided changes in treatment improve patient outcomes. It only estimates potential net benefit under assumed threshold probabilities. Therefore, the DCA results should be interpreted as supportive evidence for exploratory clinical utility rather than proof of direct clinical applicability.

The potential clinical value of preoperative MVI prediction lies in risk stratification before treatment. The intended clinical setting of the present nomogram is exploratory preoperative MVI risk stratification in surgically treated or potentially resectable HCC patients with clinical characteristics broadly similar to those of the study cohorts. These cohorts were predominantly male and HBsAg-positive, with a high prevalence of background cirrhosis, preserved liver function, and early-stage or resectable disease, reflecting the epidemiological profile of HCC in southern China. MVI has been associated with postoperative recurrence, and previous studies have suggested that anatomical resection or wider surgical margins may be associated with improved oncological outcomes in selected HCC patients with MVI or high MVI risk (28, 29). In this context, the current nomogram may provide supportive information for multidisciplinary discussion regarding resection extent, surgical margin, liver functional reserve, and postoperative surveillance. However, treatment strategy cannot be determined by predicted MVI risk alone. Surgical decision-making must also consider tumor location, future liver remnant, cirrhosis severity, portal hypertension, vascular anatomy, technical feasibility, and patient comorbidity. Thus, the appropriate role of this nomogram is to provide supportive preoperative risk information, not to mandate anatomical resection or any specific intervention.

The relationship between tumor size and MVI risk is also clinically relevant, as larger tumor size and greater tumor burden have been associated with an increased likelihood of vascular invasion in HCC (30, 31). In this study, MVI-positive tumors were larger than MVI-negative tumors in the training cohort, which is consistent with this established association. However, tumor size was not retained in the final LASSO-based multivariable model, whereas irregular tumor margins and AFP positivity remained independently associated with MVI. Irregular or non-smooth tumor margins on preoperative imaging have been shown to be associated with MVI and may reflect infiltrative growth at the tumor-liver interface (32, 33). Elevated AFP has also been reported as a clinically relevant marker associated with MVI risk and aggressive tumor biology in HCC (30, 34). Biologically, this finding suggests that morphological infiltrative patterns at the tumor-liver interface and serological tumor activity may capture intrinsic tumor invasiveness more effectively than mere physical expansion. To address potential concerns regarding the influence of tumor burden, we conducted a subgroup analysis stratified by a clinical cutoff of 5 cm. The results demonstrated that our combined nomogram maintained consistent and comparable discriminatory performance in both small (≤ 5 cm) and large (> 5 cm) HCC subgroups across both the training and validation cohorts. This finding suggests that the model’s predictive ability is robust and not substantially driven by baseline tumor size. Nevertheless, the incremental value of this two-variable model beyond conventional clinical assessment remains limited. Future studies should evaluate its performance in subgroups stratified by tumor size, tumor stage, liver function, and treatment strategy.

Several limitations should be acknowledged. First, although an independent external validation cohort was included, the retrospective design remains susceptible to selection bias and unmeasured confounding. Second, the model showed fair-to-acceptable discrimination rather than high predictive accuracy, so it should not be used as a stand-alone tool for treatment selection. Third, AFP-L3 and PIVKA-II were not routinely tested during the study period and could not be incorporated into the current model, limiting direct comparison with biomarker-enhanced models. Fourth, CT imaging features were based on qualitative visual assessment. Although interobserver agreement was acceptable, multicenter standardization is still required. Fifth, this study did not evaluate whether model-informed decisions changed surgical strategies or improved recurrence and survival outcomes. Sixth, the study population was predominantly male and HBsAg-positive, with a high prevalence of background cirrhosis, Child-Pugh class A liver function, and early-stage or resectable disease. This may limit the generalizability of the nomogram to female patients, non-HBV-related HCC, non-cirrhotic HCC, decompensated cirrhosis, advanced-stage disease, and patients undergoing non-surgical treatments. In particular, the performance of AFP positivity in HCV-related, alcohol-related, MASLD-associated, or MASH-associated HCC remains uncertain and requires further validation. Future prospective multicenter studies should compare this simple CT-AFP nomogram with radiomics, deep-learning, MRI-based, and biomarker-integrated models, assess calibration across different etiological and clinical subgroups, and determine whether preoperative MVI risk stratification improves clinically meaningful outcomes.

## Conclusion

This study developed and externally validated a preoperative nomogram combining CT imaging features and AFP levels for predicting MVI in patients with HCC. Irregular tumor margins and AFP positivity were identified as independent predictors and were incorporated into the final two-variable nomogram, whereas capsular interruption and intratumoral hyperplastic vessels were associated with MVI-related imaging characteristics but were not retained as independent predictors after adjustment. The model showed fair-to-acceptable discrimination and good calibration in the available cohorts, suggesting that it may serve as an exploratory tool for preoperative MVI risk stratification. Its potential role is to provide supportive information for multidisciplinary discussion rather than to directly determine surgical strategy, and further prospective multicenter studies are required to confirm whether model-informed risk stratification improves clinical outcomes.

## Data Availability

The original contributions presented in the study are included in the article/**Supplementary Material**. Further inquiries can be directed to the corresponding authors.
