# Correction: Development and validation of a nomogram based on tumor margin irregularity and alpha-fetoprotein for predicting microvascular invasion in hepatocellular carcinoma

**DOI:** 10.3389/fonc.2026.1970892

**Published:** 2026-09-03

**Authors:** Xiang Huang, Xiaofeng Chen, Xiangguang Chen, Ruibin Huang, Zhuozhi Dai, Ruyao Zhuang, Zhiqi Yang

**Affiliations:** 1 Department of Radiology, Meizhou People’s Hospital, Meizhou, China; 2 Department of Radiology, First Affiliated Hospital of Shantou University Medical College, Shantou, China; 3 Department of Radiology, Shantou Central Hospital, Shantou, Guangdong, China

**Keywords:** alpha-fetoprotein, computed tomography, hepatocellular carcinoma, microvascular invasion, nomogram

There was a mistake in the **Materials and methods**, *Patients* section as published. The end date of data collection for the external validation cohort was incorrectly reported.

The published text was:

“To evaluate the generalizability of the proposed predictive model, an independent external validation cohort comprising 256 patients (95 MVI-positive and 161 MVI-negative) was retrospectively established from two independent institutions (First Affiliated Hospital of Shantou University Medical College and Shantou Central Hospital) between January 2018 and December 2023.”

The correct text is:

“To evaluate the generalizability of the proposed predictive model, an independent external validation cohort comprising 256 patients (95 MVI-positive and 161 MVI-negative) was retrospectively established from two independent institutions (First Affiliated Hospital of Shantou University Medical College and Shantou Central Hospital) between January 2018 and December 2024.”

There was also a mistake in **Figure 1** as published. In the patient enrollment flow diagram, the end date of the external validation cohort from the First Affiliated Hospital of Shantou University Medical College and Shantou Central Hospital was incorrectly displayed as December 2023. The correct end date should be December 2024. The corrected **Figure 1** appears below.

**Figure 1:**
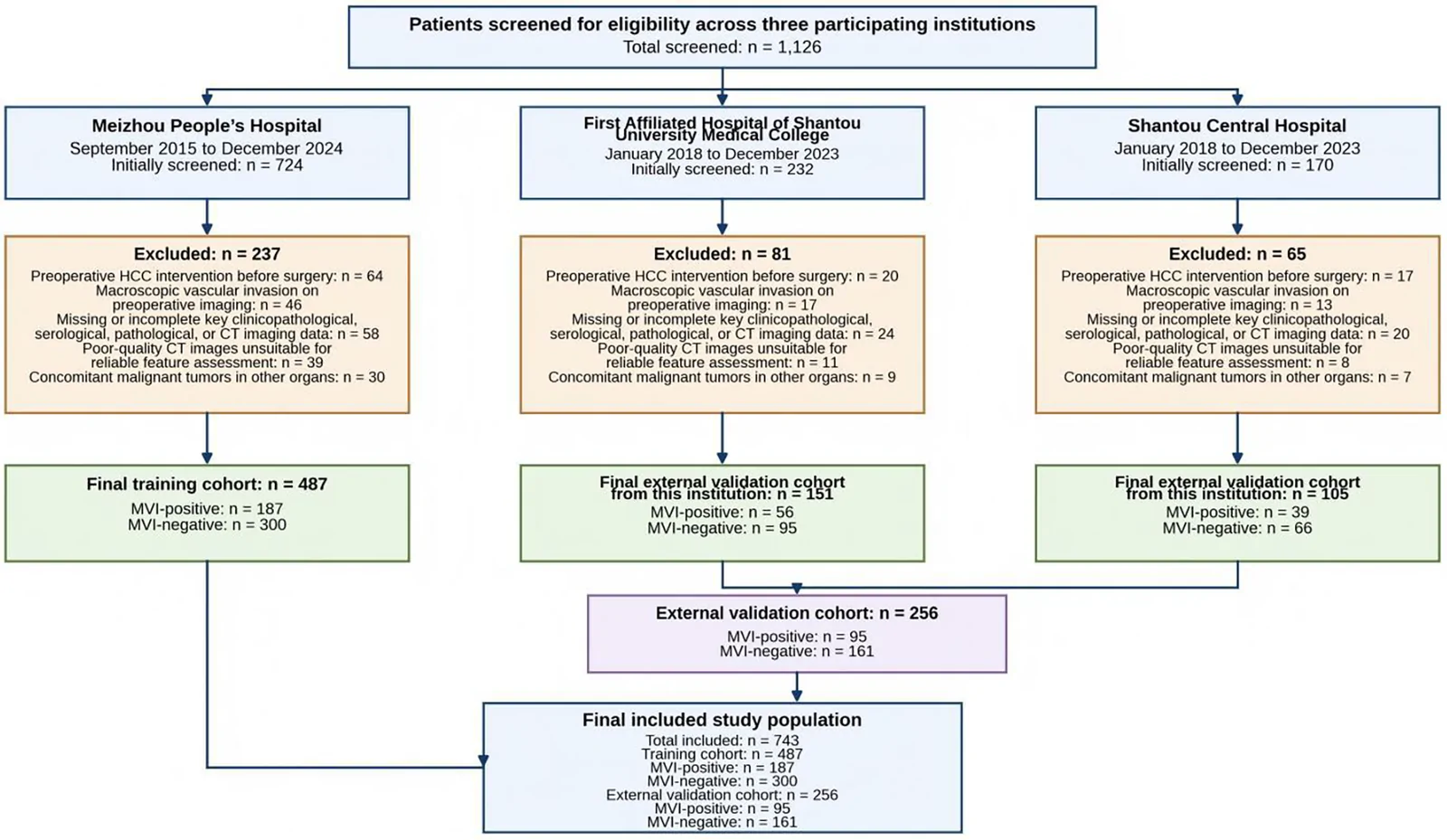
Patient enrollment flow diagram. The diagram shows the number of patients initially screened at each participating institution, the number excluded according to each predefined exclusion criterion, and the final numbers included in the training and external validation cohorts stratified by microvascular invasion (MVI) status. HCC, hepatocellular carcinoma; CT, computed tomography; MVI, microvascular invasion.

There was also a mistake in **Figure 3** as published. In the MVI-negative HCC case shown in panels E–H, the tumor annotation was placed at an incorrect location. The underlying CT images and imaging interpretation remain unchanged. Only the annotation indicating the tumor location has been corrected. The corrected **Figure 3** appears below.he original version of this article has been updated.

**Figure 3:**
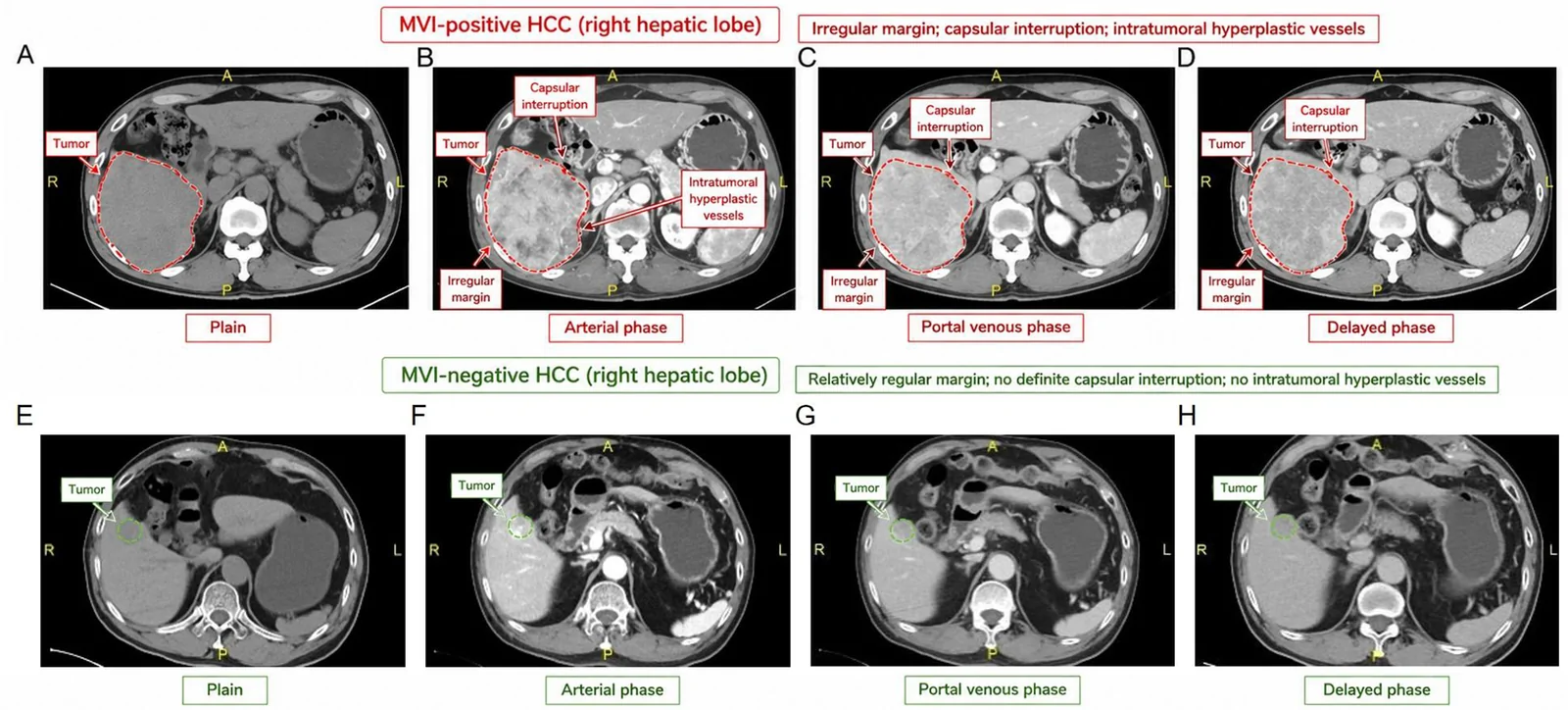
Representative dynamic contrast-enhanced CT images contrasting typical preoperative imaging features between MVI-positive and MVI-negative hepatocellular carcinoma (HCC) patients. **(A–D)** MVI-positive HCC (alpha-fetoprotein [AFP] ≥ 400 ng/mL) in the right hepatic lobe. **(A)** The plain scan shows a heterogeneous low-density mass. **(B–D)** Dynamic enhancement phases highlight classical high-risk imaging features: an irregular tumor margin (red dashed outline), discernible capsular interruption, and the presence of intratumoral hyperplastic vessels (red arrows). These features were more frequently observed in MVI-positive tumors and were selected during the LASSO-based feature selection process. **(E–H)** MVI-negative HCC (AFP < 400 ng/mL) in the right hepatic lobe. **(E)** The plain scan reveals a more homogeneous low-density mass. **(F–H)** In contrast to the MVI-positive case, this tumor demonstrates relatively regular margins (green dashed outline), an intact capsule without definite interruption, and an absence of evident intratumoral hyperplastic vessels. Both cases illustrate the characteristic “wash-in and wash-out” enhancement pattern typical of HCC. HCC, hepatocellular carcinoma; MVI, microvascular invasion.

