# Supplementary material for: Development and validation of a nomogram based on tumor margin irregularity and alpha-fetoprotein for predicting microvascular invasion in hepatocellular carcinoma
Source: Front Oncol. 2026 Jun 26;16:1821034. doi: 10.3389/fonc.2026.1821034 (PMC13349876; doi:10.3389/fonc.2026.1821034)
Supplement: Supplementary file 1 [file Table1.docx]

**Supplementary Table S1.** Overall baseline comparability between the training cohort and external validation cohort

| **Variables** | **Training cohort (n = 487)** | **External validation cohort (n = 256)** | **P value** |
| --- | --- | --- | --- |
| Demographic and clinical features |  |  |  |
| Age, years, mean ± SD | 56.4 ± 10.2 | 56.7 ± 10.2 | 0.696 |
| Male sex, n (%) | 401 (82.3) | 208 (81.3) | 0.713 |
| HBsAg positive, n (%) | 410 (84.2) | 218 (85.2) | 0.729 |
| Anti-HCV positive, n (%) | 23 (4.7) | 10 (3.9) | 0.608 |
| Cirrhosis, n (%) | 374 (76.8) | 201 (78.5) | 0.595 |
| Child-Pugh class, n (%) |  |  | 0.535 |
| Class A | 461 (94.7) | 245 (95.7) |  |
| Class B | 26 (5.3) | 11 (4.3) |  |
| PLT, ×10⁹/L, mean ± SD | 168.7 ± 60.0 | 165.7 ± 61.7 | 0.517 |
| TBIL, μmol/L, median (IQR) | 14.0 (9.9–18.2) | 14.1 (10.2–18.4) | 0.684 |
| Tumor-related characteristics |  |  |  |
| Tumor number, n (%) |  |  | 0.918 |
| Solitary | 421 (86.4) | 222 (86.7) |  |
| Multiple | 66 (13.6) | 34 (13.3) |  |
| BCLC stage, n (%) |  |  | 0.577 |
| Stage A | 437 (89.7) | 233 (91.0) |  |
| Stage B | 50 (10.3) | 23 (9.0) |  |
| Edmondson-Steiner grade, n (%) |  |  | 0.905 |
| Grade I–II | 335 (68.8) | 175 (68.4) |  |
| Grade III–IV | 152 (31.2) | 81 (31.6) |  |
| Serological indices |  |  |  |
| PLR, mean ± SD | 140.9 ± 82.5 | 141.3 ± 94.9 | 0.954 |
| Albumin, g/L, mean ± SD | 39.7 ± 5.6 | 40.8 ± 4.5 | 0.003 |
| Globulin, g/L, mean ± SD | 29.7 ± 7.3 | 30.4 ± 5.2 | 0.147 |
| ALT, U/L, median (IQR) | 42.0 (26.0–76.0) | 41.5 (26.0–73.0) | 0.682 |
| AST, U/L, median (IQR) | 43.0 (27.0–67.0) | 48.0 (31.0–74.0) | 0.081 |
| ALP, U/L, median (IQR) | 93.0 (70.0–127.0) | 101.0 (76.0–149.0) | 0.067 |
| Tumor markers |  |  |  |
| AFP, n (%) |  |  | 0.148 |
| Negative (<400 ng/mL) | 182 (37.4) | 82 (32.0) |  |
| Positive (≥400 ng/mL) | 305 (62.6) | 174 (68.0) |  |
| CEA, n (%) |  |  | 0.003 |
| Negative (<5 ng/mL) | 397 (81.5) | 230 (89.8) |  |
| Positive (≥5 ng/mL) | 90 (18.5) | 26 (10.2) |  |
| CA199, n (%) |  |  | 0.458 |
| Negative (<37 U/mL) | 456 (93.6) | 236 (92.2) |  |
| Positive (≥37 U/mL) | 31 (6.4) | 20 (7.8) |  |

Notes: This supplementary table compares the overall baseline characteristics between the training cohort and the external validation cohort, regardless of MVI status. Continuous variables conforming to a normal distribution are presented as mean ± standard deviation and were compared using the independent samples t-test. Non-normally distributed variables are presented as median (interquartile range) and were compared using the Mann-Whitney U test. Categorical variables are presented as number (percentage) and were compared using the chi-square test or Fisher exact test, as appropriate. MVI, microvascular invasion; HBsAg, hepatitis B surface antigen; HCV, hepatitis C virus; PLT, platelet count; TBIL, total bilirubin; BCLC, Barcelona Clinic Liver Cancer; PLR, platelet-to-lymphocyte ratio; ALT, alanine aminotransferase; AST, aspartate aminotransferase; ALP, alkaline phosphatase; AFP, alpha-fetoprotein; CEA, carcinoembryonic antigen; CA199, carbohydrate antigen 199; SD, standard deviation; IQR, interquartile range.

**Supplementary Table S2.** Sensitivity analysis after incorporating tumor size into the final predictive model

| **Model** | **Predictor** | **β** | **OR** | **95% CI** | **P value** | **AUC** | **95% CI for AUC** | **DeLong P vs final model** |
| --- | --- | --- | --- | --- | --- | --- | --- | --- |
| Final model | Irregular margins | 1.663 | 5.275 | 3.165–8.791 | <0.001 | 0.740 | 0.696–0.784 | Reference |
|  | AFP ≥400 ng/mL | 1.193 | 3.297 | 1.983–5.481 | <0.001 |  |  |  |
| Tumor size-adjusted sensitivity model | Irregular margins | 1.582 | 4.864 | 2.894–8.176 | <0.001 | 0.748 | 0.705–0.791 | 0.218 |
|  | AFP ≥400 ng/mL | 1.151 | 3.162 | 1.892–5.285 | <0.001 |  |  |  |
|  | Maximum tumor diameter, per 1-cm increase | 0.043 | 1.044 | 0.992–1.098 | 0.096 |  |  |  |

Notes: The tumor size-adjusted sensitivity model was constructed by forcing maximum tumor diameter into the final two-variable model. Tumor size was entered as a continuous variable per 1-cm increase. The final model included irregular tumor margins and AFP positivity. AFP, alpha-fetoprotein; OR, odds ratio; CI, confidence interval; AUC, area under the curve.
